# Pulmonary hemorrhagic syndromes

**DOI:** 10.1002/ppul.27250

**Published:** 2024-09-11

**Authors:** Andrew Bush

**Affiliations:** ^1^ National Heart and Lung Institute, Imperial College, and Imperial Centre for Paediatrics and Child Health London UK; ^2^ Department of Paediatric Respiratory Medicine Royal Brompton Hospital London UK

**Keywords:** autoimmune, celiac disease, idiopathic pulmonary hemosiderosis, pulmonary vasculitis

## 1 INTRODUCTION

There are numerous causes of bleeding from the lungs, the commonest of which are airway diseases. The pulmonary hemorrhagic syndromes are the of genetic, acquired and unknown causes, and a logical approach to diagnosis is necessary.^1^ Causes of bleeding which may cause diagnostic confusion include factitious, and sources in the upper airway and gastrointestinal tract. Iatrogenic trauma, such as erosion of an endotracheal tube should not be a diagnostic problem. True pulmonary hemorrhage may be from the bronchial arterial tree, or alveolar in origin. The Table 1 summarizes the major differential diagnoses of the pulmonary hemorrhagic syndromes.

**Table 1:** Differential diagnosis of pulmonary hemorrhagic syndromes.

Infection: Bronchiectasis (any cause, usually severe and clinically obvious airway disease), bronchitis, lung abscess, pneumonia, tuberculosis. Trauma: Airway laceration, lung contusion, artificial airway, suction catheters, foreign body, inhalation injury. Vascular Disorders: Pulmonary embolism/thrombosis, pulmonary arteriovenous malformations including hereditary hemorrhagic telangiectasia, hemangioma, bronchial Dieulafoy's disease, malignancy, pulmonary hypertension, cardiac disease. Coagulopathy: Von Willebrand's disease, thrombocytopenia, iatrogenic due to anticoagulants, other medications. Congenital Lung Malformations: Any. Miscellaneous: Catamenial, neoplasm. Inhalational injury: Vaping.

## 2 PULMONARY (ALVEOLAR) HEMORRHAGIC SYNDROMES

These are rare, with an estimated prevalence of 0.24–1.23 cases/million children/year in National studies. The presentation in childhood may be subtle. Hemoptysis may not be a feature and presentation may be nonspecific, including breathlessness, fatigue and iron deficiency anemia. Indeed, many children undergo unnecessary invasive investigations for gastroenterological causes of anemia before the lungs are identified as the cause. Occasionally, presentation may be dramatic and acute. Physical signs may include digital clubbing, pallor, tachypnoea and respiratory distress, and signs of extrapulmonary involvement. Imaging (chest X‐ray (CXR), computed tomography (CT)) shows diffuse infiltrates but is nonspecific. Confirmation that pulmonary bleeding has occurred is by bronchoalveolar lavage (BAL), demonstrating hemosiderin laden macrophages, but this does not allow a specific diagnosis to be made. Serological investigations will include platelet count and coagulation studies, an autoantibody screen, celiac screen to exclude Lane‐Hamilton syndrome and an echocardiogram to exclude cardiac causes. The question of lung biopsy is debated. A biopsy may allow the diagnosis of neutrophilic pulmonary capillaritis, but there is often poor agreement on this diagnosis even between specialist pediatric pathologists. A biopsy may suggest a specific diagnosis (below).

Broadly, the causes of alveolar hemorrhagic syndromes can be considered as Idiopathic pulmonary hemosiderosis (IPH, a diagnosis of exclusion); isolated pulmonary vasculitis; pulmonary vasculitis as part of a systemic disease; and other systemic causes of diffuse alveolar hemorrhage (DAH). Heiner's syndrome (IgD milk antibodies, now nowhere measured) is vanishingly rare, if indeed it exists. There are no randomized controlled trials of treatment; IPH is generally treated with systemic corticosteroids, often intravenous pulsed methyl prednisolone, sometimes with additional azithromycin and hydroxychloroquine. For refractory cases, other immunosuppressants such as methotrexate, azathioprine and rituximab have been prescribed on an anecdotal basis. Pulmonary vasculitis is treated with more intensive induction regimes, including combined pulses of methyl prednisolone, cyclophosphamide and immunoglobulin, sometimes combined with plamapheresis, again based on anecdotal evidence.

## 3 RECENT LARGE SERIES

Two recent big series have helped our understanding of these rare syndromes. A pan‐European collaboration recruited 117 patients from 26 centers in 15 countries, of whom 53 were ≥6 years old at diagnosis.^2^ Clinical presentation was with anemia (87%), haemoptysis (42%), dyspnea (35%) and cough (32%). Importantly, respiratory symptoms were absent in 26%. Diagnostic workup included CT (94%), BAL (85%) and echocardiography (80%). Lung biopsy was performed in 49 (42%) and at least some genetic testing in 52 (44%). Rheumatology serology was performed in in 97 patients (83%), of whom 44 (45%) tested positive for one or more autoantibodies. Anti‐transglutaminase IgA and cow's milk IgG was elevated in 17% and 14% respectively of those tested. Lung function testing when performed was restrictive; median (IQR) %pred first second forced expired volume (FEV_1_) (66% [56;86]), forced vital capacity (FVC) (68% [56;86]) with FEV_1_/FVC (96% [84;106]). Total lung capacity (TLC) was (87% [72;106]) and carbon monoxide transfer (DLCO) (69% [58;91]). Reported diagnoses were idiopathic pulmonary hemosiderosis (*n* = 35), DAH associated with autoimmune features (*n* = 20), systemic and collagen disorders (*n* = 18), immuno‐allergic conditions (*n* = 10), other interstitial lung diseases (*n* = 5), autoinflammatory diseases (*n* = 3), DAH secondary to other conditions (*n* = 21) and unspecified DAH (*n* = 5). The most frequent medical treatment was systemic corticosteroids (93%), hydroxychloroquine (35%) and azathioprine (27%). The median (IQR) time of follow‐up was 4 (1.5;7.8) years. The most frequent medical treatment was systemic corticosteroids (93%) followed by hydroxychloroquine (35%) and azathioprine (27%). Follow up was for a (median [IQR]) 3.2 (1.2;7.0) years from diagnosis. FVC and TLC improved by 21% and 17% (*p* < .001), respectively. Follow up CT or CXR was performed in 101 patients (86%) with a median (IQR) time of 2.5 (1.0;7.0) years after the baseline radiology. There were persisting abnormal radiology in 61/95 (64%) of patients (50% of patients otherwise considered healthy; 73% of patients considered as chronic but not on medical treatment and 80% of patients with on‐going treatment). Fifteen patients died, of the rest, 19% were considered healthy, 16% chronic clinically stable; 31% active clinical stable and 12 were unstable and having on‐going treatment.

Another large collaborative study^3^ underscored both the rareness of DAH and the fact that a thorough workup often results in a specific diagnosis (97/131 [74%]). *N* = 34 (26%), were termed IPH, of whom 20 could be assigned to descriptive clusters: IPH associated with autoimmune features (*N* = 9), eosinophilia (*N* = 5), renal disease (*N* = 3) or multiorgan involvement (*N* = 3). This group proposed a novel diagnostic algorhythm using Phenomizer https://compbio.charite.de/phenomizer/. The key message from both studies is that a diagnostic workup will yield a specific diagnosis in many cases.

## 4 SOME SPECIFIC CONDITIONS

Alveolar bleeding may be part of an antineutrophil cytoplasmic antibody–associated vasculitis, including microscopic polyangiitis (12%–55%) and in 7%–45% of patients with granulomatosis with polyangiitis (7%–45%, formerly known as Wegener's granulomatosis). Other causes include anti‐glomerular‐basement membrane disease (Goodpasture syndrome); systemic lupus erythematosus (rare, 2.5% but can be life‐threatening); Henoch‐Schönlein purpura; Behçet's disease; cryoglobulinemic vasculitis; and juvenile idiopathic arthritis. Recently described genetic causes include STING‐associated vasculopathy with onset in infancy (SAVI, an interferonopathy)^4^ and coatomer protein complex (COPA) disease. These are important because there are relatively specific and effective treatments: corticosteroids and JAK inhibitors (SAVI) and corticosteroids, cyclophosphamide and rituximab (COPA).^5^

## 5 CONCLUSIONS

The alveolar hemorrhagic syndromes are rare and may be difficult to diagnose because the presentation may be subtle. Extrapulmonary and airway causes of bleeding should be excluded. A specific diagnosis should be aggressively pursued because there may be specific treatments for some entities. Severe acute DAH carries a bad prognosis.

## AUTHOR CONTRIBUTIONS

**Andrew Bush**: Investigation; conceptualization; writing—original draft; writing—review and editing.

## CONFLICT OF INTEREST STATEMENT

The author declares no conflicts of interest.

## Data Availability

Data sharing is not applicable to this article as no new data were created or analyzed in this study.
